# Breaking the bad: *Bacillus* blocks fungal virulence factors

**DOI:** 10.15698/mic2017.11.599

**Published:** 2017-10-30

**Authors:** François L. Mayer, James W. Kronstad

**Affiliations:** 1 Michael Smith Laboratories, Department of Microbiology and Immunology, University of British Columbia, Vancouver, British Columbia, Canada.

**Keywords:** melanin, capsule, biofilm, filamentation, chitin, chitinase

## Abstract

Fungal pathogens rely on the production of specific virulence factors during infection. Inhibiting such factors generally results in reduced fungal pathogenicity. Most studies in the past have focused on understanding the molecular mechanisms of fungal virulence factor expression during mono-culture, or during interaction with the host. However, a potentially important, second type of interaction has been less well studied thus far - the interplay of fungal pathogens of humans with other microbes found in their natural habitat. Specifically, whether environmental bacteria may impact fungal virulence factor production is largely unknown. In our recent work, we have identified the soil bacterium, *Bacillus safensis*, as a potent inhibitor of virulence factor production by two major fungal pathogens of humans, *Cryptococcus neoformans*, and *Candida albicans*. We determined that the anti-virulence factor mechanism is, at least in part, based on production of bacterial chitinases that target and destabilize the fungal cell surface. These findings describe a cross-kingdom interaction between an environmental bacterium and pathogenic fungi, and highlight the fungal cell wall as an attractive antifungal drug target.

Fungal pathogens pose a largely under-appreciated threat to human health. The AIDS-associated, pathogenic fungus *C. neoformans*, for example, causes ~223,000 new infections each year with a mortality rate exceeding 80%. Only a few potent antifungal drugs exist to treat these infections. *C. neoformans* is found globally, with particularly high prevalence in Africa, India, China, Indonesia, and North America. Its natural habitat is the environment: *C. neoformans* lives in soil, on trees, and in bird droppings. Infection of humans occurs through inhalation of spores or desiccated yeast cells. In immunocompromised individuals, the fungus can cause an opportunistic pulmonary infection. Importantly, *C. neoformans* has the capacity to disseminate from the lungs to the brain and cause life-threatening meningoencephalitis in patients with severe immune system deficiencies, such as persons with AIDS.

To initiate and maintain an infection, *C. neoformans* employs a range of different virulence factors, including production of the black pigment melanin, synthesis of a polysaccharide capsule, and secretion of hydrolytic enzymes. Melanin is synthesized from *o*-diphenolic compounds, such as 3,4-dihydroxyphenylalanine (L-dopa), and is anchored to the chitin layer of the fungal cell wall, or is secreted into the environment. The pigment has antioxidant activity, and mutants with defects in melanin biosynthesis are usually strongly reduced in virulence. The polysaccharide capsule is also associated with the fungal cell surface; it is assembled onto the (&-1,3-glucan layer of the fungal cell wall. The polysaccharide capsule protects the fungus from attack by human immune cells, primarily macrophages, and has immunomodulatory properties. Inhibiting capsule formation genetically results in strongly reduced fungal virulence. Strikingly, capsule was also demonstrated to protect *C. neoformans* from attack by amoebae, which may act as natural fungal predators. Hydrolytic enzymes produced by *C. neoformans* include urease and phospholipases, and these enzymes contribute to virulence as well. Furthermore, *C. neoformans* has the capacity to form drug-recalcitrant biofilms consisting of fungal cells encased in an extracellular matrix of capsule polysaccharides. Most research efforts in the past have focused on understanding the specific roles these virulence factors play during host-pathogen interaction. However, few studies have addressed another type of interaction that likely (and probably predominantly) occurs: the cross-kingdom interplay between *C. neoformans* and environmental bacteria, and its potential impact on fungal virulence factor production.

In our recent work, we addressed the lack in knowledge about cross-kingdom interactions by isolating and characterizing bacteria from natural cryptococcal niches including soil and plants. By performing dual-species incubation experiments on L-DOPA medium, we discovered that several bacteria of the genus *Bacillus* blocked the capacity of *C. neoformans* to produce melanin. One bacterium in particular, *B. safensis*, had strong anti-melanization activity (Fig. 1A). This inhibitory effect was dependent on both cell-cell contact and bacterial viability. Indeed, bacteria appeared to actively swarm around fungal colonies to establish close proximity (Fig. 1A). By screening a collection of mutants defective in transcription factors, and testing exogenous enzymes and inhibitors, we implicated chitinase activity at the fungal cell wall as a component of the inhibitory mechanism. The anti-melanization activity of *B. safensis* was not limited to *C. neoformans*, but also extended to another, closely related species, *Cryptococcus gattii*. This species is interesting because it is currently causing an outbreak of cryptococcal disease in immunocompetent people in British Columbia, and we included soil known to be positive for *C. gattii* in our study.

**Figure 1:**
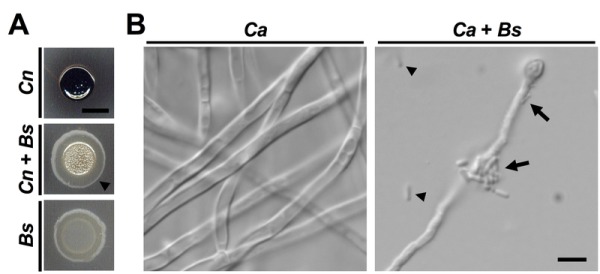
FIGURE 1: *B. safensis* inhibits virulence factor production by pathogenic fungi. **(A)** The *C. neoformans *wild type strain H99, *B. safensis*, or a mixture of both were spotted onto L-DOPA agar and incubated at 37°C for three days before being photographed. Fungal cells alone melanize resulting in a black colony color. During dual-species interaction, *B. safensis *blocks cryptococcal melanization resulting in light beige-colored colonies. Arrowhead points to bacterial swarming. *Cn*, *C. neoformans*; *Bs*, *B. safensis*. Scale bar, 5 mm. **(B)** The *C. albicans *wild type SC5314 was incubated under filament-inducing conditions (10% fetal calf serum, 37°C, 5% CO_2_) for 24 h with or without *B. safensis*. Fungal filamentation was then analyzed by DIC microscopy. Arrowheads indicate free-floating bacterial cells, while arrows point to bacteria that have attached to a fungal filament.* Ca*, *C. albicans*; *Bs*, *B. safensis*. Scale bar, 5 µm.

To determine whether anti-virulence factor activity of *B. safensis* was specific to the basidiomycetous fungal pathogens, we performed cross-kingdom interaction experiments with the ascomycetous fungal pathogen, *Candida albicans*. Although *C. albicans* has been reported to produce melanin under certain conditions, it is generally not considered a major virulence factor in this pathogen. The most important *C. albicans* virulence factor is the yeast-to-filament transition. We found that *B. safensis* inhibited this morphological transition and attached to fungal filaments (Fig. 1B). Further studies revealed that *B. safensis* not only inhibited fungal pigment production and morphological plasticity, but also suppressed several other important factors. Specifically, *B. safensis* inhibited polysaccharide capsule formation in *C. neoformans*, and biofilm formation in both *C. neoformans *and *C. albicans *(Fig. 2A). As mentioned earlier, further experiments revealed that the anti-virulence factor mechanism was, at least in part, based on production of one or more bacterial chitinases that target and destabilize the fungal cell surface. Consistent with this observation, we found that exogenous chitinase partially inhibited capsule formation for *C. neoformans* and filament formation for *C. albicans*.

**Figure 2:**
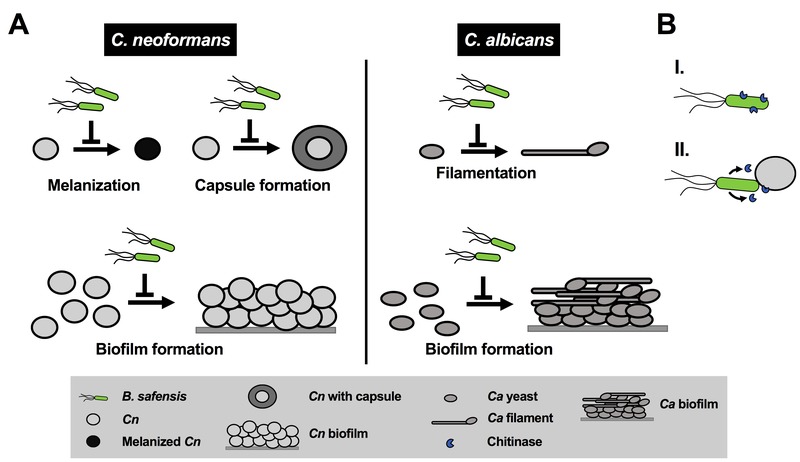
FIGURE 2: Model summarizing the inhibitory impact of *B. safensis* on virulence factor production by *C. neoformans* and *C. albicans*. **(A)** *B. safensis* inhibits melanin formation, capsule production, and biofilm formation by *C. neoformans*. During dual-species interaction with *C. albicans*, bacteria inhibit filament formation and biofilm production.* Cn*, *C. neoformans*; *Ca*, *C. albicans*. **(B)** Based on our results, we propose that destabilization of the fungal cell wall via bacterial chitinase(s) is an important factor contributing to the observed antifungal activities. *B. safensis* either produces cell surface-associated chitinase(s) (I), or, chitinase and other activities from the bacteria are only produced upon contact and are poorly diffusible (II).

Why would *B. safensis* require contact with the fungal cells to exert its antifungal effect? We speculate that one of two possible scenarios may explain this observation: first, the chitinase activity (and perhaps other activities) is associated with the bacterial cell surface and not secreted (Fig. 2B). Indeed, we did not see activity in bacterial culture supernatants. Second, it is also possible that chitinase and other activities from the bacteria are only produced upon contact or are poorly diffusible under the conditions we tested (Fig. 2B). When we exposed *C. neoformans* or *C. albicans* to exogenous chitinase (without bacteria), we observed only partial inhibition of some of the fungal virulence factors, for example, capsule production by *C. neoformans*, and filament formation by *C. albicans*. These results indicate that additional activities are likely to also contribute to the observed anti-pathogen activity. Future studies may search for these activities, examine the role of chitinases in more detail by deleting the *B. safensis* gene(s), and evaluate the impact of anti-virulence factor activity during co-incubation with fungal pathogens.

Our findings may pave the way for potential ecological and clinical applications. For example, *B. safensis* may be applied to soils with high cryptococcal burden to suppress fungal virulence factor production. In our experimental settings, *B. safenis* only had modest negative effects on overall fungal proliferation. Targeting virulence factors, rather than growth, has been proposed as an alternative approach to tackle microbial infections because of the reduced risk of resistance development. It is tempting to speculate that *B. safensis*, or its chitinase(s) could also be developed into probiotics to treat or prevent fungal infection. However, this approach would require comprehensive activity and toxicity/bioavailability studies. Overall, our findings describe a cross-kingdom interaction between a soil bacterium and pathogenic fungi, and suggest that investigating such interactions is a powerful approach for the discovery of novel antifungal drug targets, antifungal compounds and anti-virulence factors.

